# Inequality in healthcare-seeking behavior among women with pelvic organ prolapse: a systematic review and narrative synthesis

**DOI:** 10.1186/s12905-023-02367-3

**Published:** 2023-05-03

**Authors:** Melese Siyoum, Wondwosen Teklesilasie, Yitateku Alelgn, Ayalew Astatkie

**Affiliations:** 1 Department of Midwifery, College of Medicine and Health Sciences, Hawassa University, Hawassa, Ethiopia; 2 School of Public Health, College of Medicine and Health Sciences, Hawassa University, Hawassa, Ethiopia

**Keywords:** Healthcare-seeking, Pelvic floor disorder, Systematic review, Narrative synthesis

## Abstract

**Introduction:**

Pelvic organ prolapse (POP) affects women’s quality of life in various aspects. However, evidence on the healthcare-seeking behavior of women with POP is limited. Therefore, this review aimed to identify and synthesize the existing evidence on the healthcare-seeking behavior among women with POP.

**Methods:**

This systematic review and narrative synthesis of the literature on healthcare-seeking behavior among women with POP was conducted from 20 June to 07 July 2022. The electronic databases PubMed, African Journals Online, Cumulative Index to Nursing and Allied Health Literature, African Index Medicus and Directory of Open Access Journal, and Google Scholar were searched for relevant literature published from 1996 to April 2022. The retrieved evidence was synthesized using a narrative synthesis approach. The characteristics of included studies and the level of healthcare-seeking behavior were summarized in a table and texts. Error bar was used to show the variability across different studies.

**Results:**

A total of 966 articles were retrieved of which only eight studies with 23,501 women (2,683 women with pelvic organ prolapse) were included in the synthesis. The level of healthcare-seeking behavior ranges from 21.3% in Pakistan to 73.4% in California, USA. The studies were conducted in four different populations, used both secondary and primary data, and were conducted in six different countries. The error bar shows variation in healthcare-seeking behavior.

**Conclusions:**

The level of health-care seeking behavior among women with POP is low in low-income countries. There is substantial variability in the characteristics of the reviewed studies. We recommend a large-scale and robust study which will help to better understand the healthcare-seeking behavior among women with POP.

**Supplementary Information:**

The online version contains supplementary material available at 10.1186/s12905-023-02367-3.

## Introduction

Pelvic organ prolapse (POP) is a hernia of the vaginal walls (anterior and posterior), the apex of the vagina (vaginal vault), and the uterus through/into the vaginal opening [1]. Those who developed symptoms complain of a sense of pelvic pressure or bulging through the vaginal opening; and may be associated with urinary incontinence, voiding dysfunction, fecal incontinence, incomplete defecation, and sexual dysfunction [2].

The prevalence of POP varies depending on the definition utilized [3] ranging from 3 to 50%. The anatomical prevalence was found among 50% of the general women but only 3–6% of them were symptomatic [4, 5]. The prevalence is affected by different risk factors. Among others, pregnancy and vaginal delivery which may cause direct injury to pelvic floor muscles and connective tissues, and older age which causes weakness in connective tissues are the leading causes of POP [6–8]. The risk factors are both non-modifiable (family history, ethnicity, and age) and modifiable (obesity, underweight, heavy lifting, and chronic medical problems) [7, 9–12]. POP is managed either conservatively (using pessary or pelvic muscle training) for mild degree prolapse [9, 13, 14], and surgically for severe prolapse [15, 16].

Healthcare-seeking behavior is an individual’s action to promote maximum well-being, recovery, and rehabilitation [17]. It can be viewed as a person’s interaction with a certain health service. It covers details including how symptoms are viewed and handled, as well as which healthcare services are used and when [18]. In general, many interactions and relationships within supportive or restrictive social systems affect healthcare-seeking behavior [19–22].

Healthcare-seeking behavior of women with POP and other pelvic floor disorders (PFD) is unsatisfactory [23–25], as disclosing the information is associated with discrimination and the disease is considered shameful [26–30]. Women with POP do not seek treatment until the disease progresses and worsen their quality of life [31, 32]. Women’s quality of life is significantly impacted by POP [33–36]. The impact of POP on women’s quality of life manifests as sexual dysfunction, prejudice, self-consciousness, a sensation of being “less feminine,“ distraction from daily tasks, and embarrassment when requesting others’assistance with tasks like carrying heavy goods [26, 27, 37]. Consequently, many women adopt coping mechanisms and behaviors to reduce the negative effects of POP on their quality of life [38].

The evidence about healthcare-seeking behavior among POP patients is inadequate [39], as the available studies in the field of the female pelvic floor are focused on pelvic floor disorder in general [40, 41] and urinary incontinence [42–44]. Moreover, the few studies that assessed the levels of healthcare-seeking behavior among POP patients so far are inconsistent. For instance, healthcare-seeking behavior was 52–64% among POP patients in Nepal [27, 28] and 15.5–40.3% in Ethiopia [30, 38, 45]. This inconsistency makes it difficult to understand the level of healthcare-seeking behavior of women with POP.

Systematically reviewing the existing evidence, identifying the gap, and synthesizing the reported level of healthcare-seeking behavior helps to understand the burden of the problem. This has the potential to inform health professionals, policymakers, and researchers about this risk. Therefore, we conducted this systematic review to systematically identify the literature, and synthesize and narrate the available evidence on the healthcare-seeking behavior of women with POP.

## Materials and methods

### Study design and search process

This was a systematic review and narrative synthesis of the literature on healthcare-seeking behavior among women with POP. Literature that assessed the healthcare /treatment-seeking behavior of women with pelvic organ prolapse was systematically reviewed. The electronic databases PubMed, Directory of Open Access Journals, Cumulative Index to Nursing and Allied Health Literature (CINAHL), African Index Medicus, African Journals Online (AJOL)) and Google Scholar were searched for the relevant literature from 20 June to 07 July 2022. For the PubMed search, we used the following search terms: (“Utero vaginal prolapse” OR “vaginal prolapse” OR “pelvic organ prolapse”) AND (“health-seeking behavio*” OR “health behavio* " OR “treatment-seeking behavio*” OR “treatment-seeking” OR “healthcare-seeking behavio* " OR “care-seeking” OR “help-seeking” OR “health-seeking”). The reference lists of the studies were also searched for other relevant studies.

### Population, exposure and outcome (PEO)

#### Study participants

All women who were diagnosed to have pelvic organ prolapse.

#### Exposure

Any stage/degree of pelvic organ prolapse.

#### Outcome measurement

The outcome of the study was to identify the level of healthcare-seeking behavior of women with POP from studies published in reputable journals. The level of healthcare-seeking behavior was measured by the percentage of women with POP who sought healthcare either from health facility or traditional healers.

### Eligibility and selection criteria

Studies that assessed the healthcare-seeking behavior of women with POP and published in the English language were included in this systematic review and narrative synthesis. Studies published since 1996 and accessible online till 20 June / 2022 were included,. as there was no standard set of terms to describe pelvic anatomy and pelvic organ prolapse before 1996. In 1996, a team of researchers associated with the International Continence Society published “The Standardization of Terminology of Female Pelvic Organ Prolapse and Pelvic Floor Dysfunction” [46]. Qualitative studies and patient stories were excluded.

All identified articles were transferred to the EndNote X 7 reference manager and duplicates were removed. The titles and abstracts were reviewed and assessed against the inclusion criteria by two independent reviewers (MS and YA). Reasons for the exclusion of articles were marked. Eventually, the reviewers conducted a full review of the remaining articles and documents. The results of the assessment and inclusion processes were documented in the Preferred Reporting Items for Systematic Reviews and Meta-analyses (PRISMA) [47] (Supplementary file 1).

### Methods for data extraction

Two independent reviewers extracted the data from the list of eligible studies. Findings from the included studies were extracted and stored using a template prepared in Microsoft Excel. Based on the recommendation [47], the following important items were extracted from each study: authors’ name, publication year, the sample size, study design, study setting, study country, data collection method, and the results (level of healthcare-seeking behavior).

### Quality assessment

The methodological quality of the studies included in the review was critically evaluated using a quality assessment tool for prevalence studies developed by the Joanna Briggs Institute (JBI) [48, 49]. The tool contains nine items/questions with Yes, No, Unknown, and Not Applicable response options. A score of 1 is given for Yes and 0 for all other options. The scores were summed up and changed into percentages. The two authors (MS and YA) independently evaluated the quality of the studies, and the differences between the two authors were resolved by consensus. Finally, the methodological quality of the studies was categorized as high quality (score above 80%), moderate quality (between 60 − 80%), and low quality (below 60%) (Supplementary file 2).

### Data synthesis

A narrative synthesis was done by using tabulations and textual descriptions. A narrative synthesis was preferred due to the high heterogeneity of the reviewed articles. In a systematic review, when conducting a meta-analysis is not appropriate, or not possible due to different reasons like incompletely reported findings, or highly heterogeneous study characteristics (design, outcome measure, or study population), an alternative method called ‘narrative synthesis’ is recommended [50, 51]. In such a situation, the term ‘narrative’ indicates a description of study characteristics (qualitative), and the term ‘synthesis’ indicates result summarization (quantitative) [50]. In this review, primary synthesis was made through a textual description of the main outcomes of each included study. Then tabulations were used to present the characteristics of each study. Tabulations are typically used to provide information on study design, setting of the study, study population, sample size, data collection method, and outcome of the studies (level of healthcare-seeking behavior). These data were presented in different columns in the same table. Textual descriptions were used to narrate the findings based on the variability in the study population, study country, study setting, and data sources used in the individual studies. The level of healthcare-seeking behavior extracted from each study was summarized to show the range of healthcare-seeking behavior among women with POP. Error bars were also constructed using Microsoft Excel 2013 for the level of healthcare and its confidence interval to check for disparities among studies. The robustness of the synthesis was assessed by using the Best Evidence Synthesis (BES) method, in which only studies that meet minimal standards of methodological adequacy were included (Supplementary file 2).

## Results

### Identification of studies

**Fig. 1:**
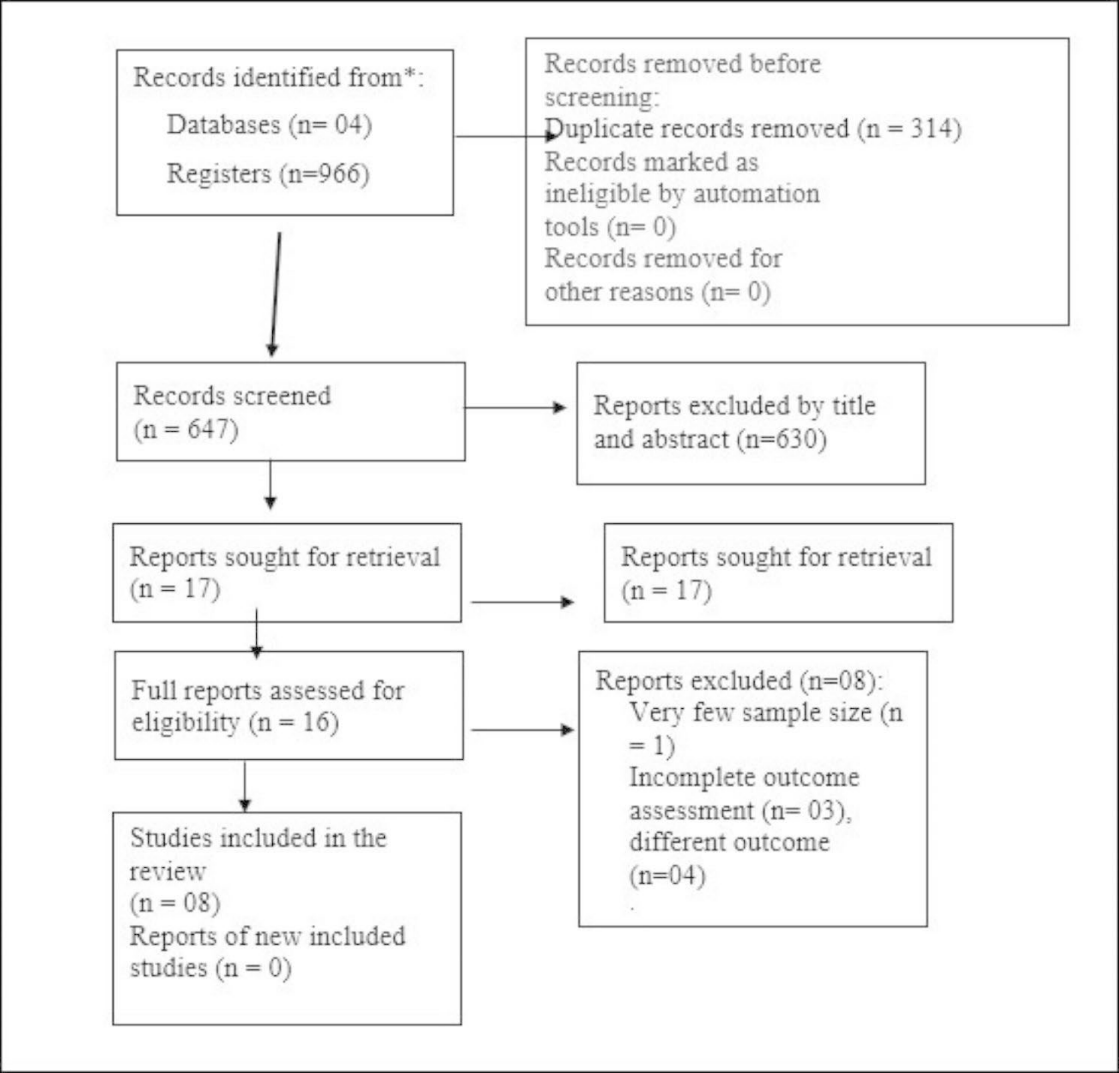
PRISMA flow diagram showing literature selection for the systematic review and narrative synthesis on healthcare seeking behaviour of patients with pelvic organ prolapse, 2022. Form obtained from (1). 1. ADDIN EN.REFLIST Page MJ, McKenzie JE, Bossuyt PM, Boutron I, Hoffmann TC, Mulrow CD, et al. The PRISMA 2020 statement: an updated guideline for reporting systematic reviews. International journal of surgery. 2021;88:105906

In this review, a total of 961 articles were identified through a search of electronic databases and references of the identified studies. Among these, 947 research articles were excluded because 314 of the studies were duplicates, 630 studies did not fulfill the eligibility criteria and one study was not freely accessible [52]. A total of 16 full-text articles were retrieved of which eight articles were excluded due to few sample size (included only seven women from rural Ghana) [53], incomplete outcome measurement [54–56], and assessment irrelevant outcome (only delay in treatment-seeking) [30, 39, 45, 57]. Finally, eight articles were included in the narrative synthesis (Fig. 1).

### Characteristics of the included studies

The studies included in the current review were published between 2007 [58] and 2020 [38]. All the eight studies included in the systematic review were cross-sectional by design; two studies were from Nepal [27, 28]; two were from the USA [59], one from the United Arab Emirates [29], one from Pakistan [60], one from Ethiopia [38] and one from Iran [61]. The sample sizes of the included studies ranged from 115 [27] to 9021 [28]. Two studies were health facility-based [27, 29] and six were community-basedstudies [38, 58, 60–62]. In general, 23,501 women (2,683 women with pelvic organ prolapse) were included in this review (Table 1).

**Table 1:** Characteristics of studies included in to the systematic review of healthcare-seeking behaviour among women with Pelvic organ prolapse, 2022

Author & year	Study Place	Study subjects	Study setting	Sample size	Data collection method	Outcome variable and results	Study period	Care-seeking for POP (%)
(Adhikari and Ranju, 2018)	Nepal	Parous women with Uterovaginal prolapse	Institution- based	9021	Secondary data	1. The prevalence of uterine prolapse = 6% 2. Help/care-seeking = 64%	2011	64
(Brazell et al., 2013)	Boston, USA	POP patients	Community-based	3205	Secondary data	Healthcare-seeking behavior across different groups for prolapse was (from total of 93 patients): • 74.2% among Black women, • 76.9% among Hispanics, and • 58.3% among white women	2002–2005	68.8
(Dheresa et al., 2020).	Ethiopia	Married women who had POP	Community-based	704	Patient Interview	Healthcare-seeking behavior was: 1. 32% of women with Pelvic Floor Disorder 2. 40.3% of POP Patients, 3. 25.9% for urinary symptoms 4. 10.4% for anal incontinence	2016	40.3
(Hammad et al., 2018)	United Arab Emirates	POP patients	Institution- based	127	Patient Interview	1. Degree of bother from POP: 111 (87.4%) had activity (physical, social, or prayers) or sexual relationship affected 2. 54% of them did not seek medical treatment	2010	46
(Jokhio et al., 2020)	Pakistan	POP patients	Community-based	5064	Patient Interview	1. Prevalence of POP: 10.3%. 2. Impact of POP: 60.8% reported quality of life is greatly or moderately affected 3. Care-seeking behavior: 78.7% never sought care	2018	21.3
(Morrill et al., 2007)	California, USA	POP patients	Community-based	4,392	Secondary data	1. Prevalence of pelvic floor disorders: • POP = 13%, urinary incontinence = 27%, • Anal incontinence = 29% and • Fecal incontinence = 19% 2. Health care-seeking behaviour: • Pelvic organ prolapse = 73% • 61% for urinary symptoms and • 28% for anal incontinence. 3. Care-seeking is associated with older age, history of hysterectomy, hormone therapy, and frequent urinary tract infection	2007	73.4
(Shrestha et al., 2014)	Nepal	POP patients	Institution- based	115	Patient Interview	1. Experience of women with POP: • 85% faced major physical discomfort, • 68% urinary symptom, • 42% bowel symptom and • 73% sexual discomfort 2. 48% never sought Healthcare	2012	52
(Tehrani et al., 2011)	Iran	Reproductive age (18-45years old) POP patients	Community-based	1252	Patient Interview	The main gynecologic morbidities were: 1. Pelvic organ prolapse (41.1%), 2. sexually transmitted infection (37.6%) and 3. Menstrual disorders (30.1%). Overall, two third of these women had not sought medical care. 4. Among the participants, 391 of them had POP whereas only 152 (39.4%) of them sought healthcare	2008–2010	39.4

* ^*POP = Pelvic organ prolapse*^

ADHIKARI, R. & RANJU, K. 2018. Uterine prolapse and treatment seeking behaviour among women. *Frontiers in Women’s Health*, 84, 7650

BRAZELL, H. D., O’SULLIVAN, D. M. & TULIKANGAS, P. K. 2013. Socioeconomic status and race as predictors of treatment-seeking behavior for pelvic organ prolapse. *American journal of obstetrics and gynecology*, 209, 476. e1-476. e5

DHERESA, M., WORKU, A., OLJIRA, L., MENGISTIE, B., ASSEFA, N. & BERHANE, Y. 2020. Women’s health seeking behavior for pelvic floor disorders and its associated factors in eastern Ethiopia. *International Urogynecology Journal*, 31, 1263–1271

HAMMAD, F. T., ELBISS, H. M. & OSMAN, N. 2018. The degree of bother and healthcare seeking behaviour in women with symptoms of pelvic organ prolapse from a developing gulf country. *BMC Women’s Health*, 18, 1–7

JOKHIO, A. H., RIZVI, R. M. & MACARTHUR, C. 2020. Prevalence of pelvic organ prolapse in women, associated factors and impact on quality of life in rural Pakistan: population-based study. *BMC women’s health*, 20, 1–7

MORRILL, M., LUKACZ, E. S., LAWRENCE, J. M., NAGER, C. W., CONTRERAS, R. & LUBER, K. M. 2007. Seeking healthcare for pelvic floor disorders: a population-based study. *American Journal of Obstetrics and Gynecology*, 197, 86. e1-86. e6

SHRESTHA, B., ONTA, S., CHOULAGAI, B., POUDYAL, A., PAHARI, D. P., UPRETY, A., PETZOLD, M. & KRETTEK, A. 2014. Women’s experiences and health care-seeking practices in relation to uterine prolapse in a hill district of Nepal. *BMC women’s health*, 14, 1–9

TEHRANI, F. R., SIMBAR, M. & ABEDINI, M. 2011. Reproductive morbidity among Iranian women; issues often inappropriately addressed in health seeking behaviors. *International Urogynecol Journal*, 11, 863

### Healthcare-seeking behavior among women with pelvic organ prolapse

Dherese et al [38] reported healthcare-seeking behavior for Pelvic Floor Disorder (PFD) in eastern Ethiopia based on a study of only married women. POP was assessed by interviewing women for symptoms of urinary incontinence, anal incontinence, and pelvic organ prolapse using the following questions: “Have you ever sought care/help for urinary symptoms (overactive bladder and/or stress urinary incontinence)?”, “Have you ever sought care/help for pelvic organ prolapse?” and “Have you ever sought care/help for anal incontinence?’. In general, 32% of women with PFDs sought health care for their problem; specifically, 40.3% of POP patients sought care, while 25.9% sought care for urinary symptoms, and 10.4% for anal incontinence.

Shrestha et al., [27] conducted a study at outreach clinics in the Dhading district in Nepal to assess women’s experience of uterovaginal Prolapse and their healthcare-seeking behavior. The report shows that 52% of 115 participants sought healthcare. Participants were included by convenience sampling at the follow-up outreach clinics.

Adhikari and Ranju [28] assessed the factors that influence the experience of uterine prolapse and the care-seeking behavior among women in Nepal. They used data extracted from the Nepal Demographic and Health Survey, 2011. They confined the analysis to only women who had one or more pregnancies. The prevalence of uterine prolapse was 6%, and only 64% of them sought treatment.

Hammed et al. [29] assessed the degree of bother, social impact, and healthcare-seeking behavior of women with symptoms of POP in the United Arab Emirates. They involved all Emirati women who attended three family development centers in the United Arab Emirates from January 2010 to January 2011. Among POP patients, 111 (87.4%) had at least one activity (physical, social, or prayers) or sexual relationship affected by POP symptoms. However, only 46% of them sought medical treatment for different reasons.

Tehrani et al. [61] evaluated reproductive morbidities and healthcare-seeking behavior of 1252 nationally representative sample of women aged 18 to 45 years in urban areas in Iran. The three main groups of morbidities they identified were: pelvic organ prolapse (41.1%), sexually transmitted infection (37.6%), and menstrual disorders (30.1%). Overall, only one-third of these women had sought medical care. Among the participants, 391 of them had POP of whom only 152 (39.4%) sought healthcare.

Morrill et al. [58] reported characteristics associated with seeking care for pelvic floor disorders (pelvic organ prolapse, urinary incontinence, and anal incontinence) in California, USA. They used secondary data on 4,392 women, which were collected for a continence-associated risk epidemiology study. Among 568 participants diagnosed to have POP (current and past history), 73% had sought treatment. Healthcare-seeking was 61% for urinary symptoms and 28% for anal incontinence. They reported that care-seeking for pelvic floor disorder is associated with older age, a history of hysterectomy, hormone therapy, and frequent urinary tract infection.

Brazell et al. [62] evaluated the prevalence of POP across a diverse group of women, and healthcare-seeking behavior among women of different ethnicities/races and socioeconomic statuses. They used secondary data collected from 3205 women for the National Institute of Health-support Boston Area community in the USA. Healthcare-seeking behavior for uterine prolapse was 74.2% among Black women, 76.9% among Hispanics, and 58.3% among White women.

Jokhio et al. [63] aimed to determine the prevalence of POP and its impact on women’s quality of life in rural Pakistan. They interviewed 5064 women and 10.3% were confirmed to have pelvic organ prolapse. Among women with POP, 60.8% reported their quality of life was greatly or moderately affected and only 21.3% of them sought care.

### Disparities in healthcare-seeking behavior among women with pelvic organ prolapse

**Fig. 2:**
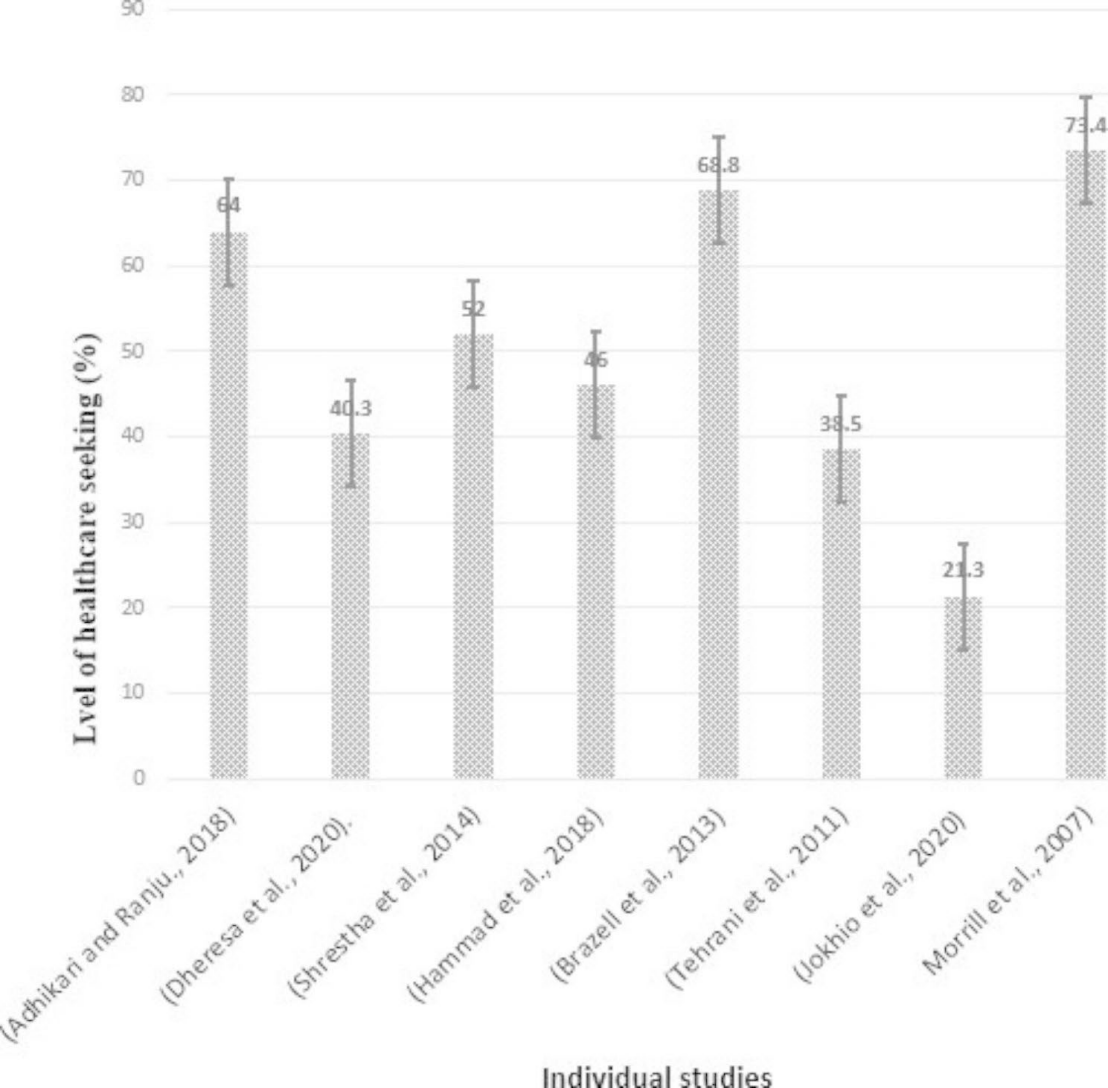
Error Bar indicating disparities in healthcare-seeking behaviour among women with Pelvic Organ Prolapse, 2022

The reported level of healthcare-seeking behavior ranges from 21.3 − 73.4%, which is 21.3% from Pakistan [60], 39.4% from Iran [61], 40.3% from eastern Ethiopia [38], 46% from United Arab Emirates [29], 52–64% from Nepal [27, 28], 68.8% from Boston, USA [62] and 73.4% from California, USA [58]. The error bar constructed for a 95% confidence interval shows disparities in healthcare seeking behavior among POP patients across settings (Fig. 2).

The reviewed studies have significant variability in terms of the study population, sample size, and study period. For example, the study from Iran included only reproductive age (18–45 years old) POP patients [61], while the study from Ethiopia involved only married women [38], and the study from Nepal involved only parous women [28] to assess their healthcare-seeking behavior. The other studies involved women who had POP. On the other hand, in three studies [28, 58, 62], health-seeking behavior analysis was done based on secondary data from National Demographic and Health Surveys, while other studies used primary data. Three studies were conducted at a health facility [27–29] while five studies were conducted at the community level (Table 1).

### Quality of included studies

Accordingly to the Joanna Briggs Institute (JBI) [48, 49] checklist for prevalence study, five studies were of high methodological quality (score > 80%) [28, 38, 61–63], and three studies were of moderate methodological quality (quality score 60–80%) [27, 29, 58] (Supplementary file 2).

## Discussion

This systematic review presents a summary of healthcare-seeking behavior in women with pelvic organ prolapse. The level of healthcare-seeking behavior of women with POP is not uniform; it ranges from 21.3% [63] to 73.4% [58]. There was also substantial disparity in healthcare-seeking behavior among POP patients across settings.

The review revealed that there is a disparity in the level of healthcare-seeking behavior. The observed variation might be due to the difference in the characteristics of the studies. The studies included in the present review are highly heterogeneous in terms of the study population, study setting, study period, and sample size. For example, some studies involved only married women with POP [38], some others involved only parous women [28] and others involved all women with POP [29, 57, 58]. This difference in characteristics of the study subject might be one source of difference in healthcare-seeking behavior. For instance, married women may have spousal support and seek healthcare more than the unmarried groups. This was evidenced by a study from southern Ethiopia which identified lack of support as one of the determinant factors for not seeking healthcare [45]. Two studies from Jamaica and South Africa showed that healthcare-seeking behavior is higher among married people [64, 65].

In this review, the lowest level of healthcare-seeking behavior was reported in Pakistan (21.3%) [63] and the highest level was reported in California, USA (73.4%) [58]. The low level of healthcare seeking in Pakistan was justified to be due to a lack of awareness about the availability of treatment and the high cost of care at that time [63]. The healthcare-seeking reports from eastern Ethiopia (40.3%) [38] and Iran (39.4%) [61] were higher than the healthcare-seeking behavior report from Pakistan (21.3%). The difference could be due to differences in the inclusion criteria to recruit participants. The study from eastern Ethiopia involved only married women and the study from Iran involved only reproductive age (18–45 years) women, while the study from Pakistan involved all women. Reproductive age and married women can get support and access to money compared to their counterparts. A systematic review and meta-analysis conducted on barriers and promoting factors to health service utilization for pelvic floor disorder in the United States showed that having social support and established primary/secondary care were facilitating factors for care seeking [66].

In this review, the level of healthcare-seeking behavior reports from Boston, USA (68.8%) [62] and California, USA (73.4%) [58], are higher than the health-seeking behavior reported in Ethiopia (40%), Iran (39.4%), Nepal (52%), United Arab Emirate (46%) and Pakistan (21%). Among other reasons, this could be related to differences in economic status, availability of health care, and fear of stigma. It was repeatedly reported that the common reasons for not seeking healthcare for POP in low-income countries were lack of money for transportation and healthcare payment [28, 30, 45, 67], and fear of social discrimination [38]. Two studies from eastern Ethiopia and Iran showed that a moderate wealth index and financial support improved health seeking behavior of women with pelvic floor disorders [68, 69]. In addition, lack of awareness about the pelvic floor and experiencing fear were barriers to care-seeking [66, 68, 69]. A systematic review conducted in the United States showed that health service utilization for pelvic floor disorder was low among Black women [66]. In low-income countries, even among women who seek health care, most of them were delayed for more than a year before seeking care. Among women admitted to hospitals for POP surgery, 82% in Ethiopia [30] and 36% in Uganda [57] were delayed at least 12 months before seeking care.

To our knowledge, this is the first systematic review and narrative synthesis that assessed the healthcare-seeking behavior of women with POP from all countries. The review has synthesized and revealed the level of healthcare-seeking behavior in various settings based on the best available evidence. The primary studies included in this review were critically assessed for methodological quality. The possible source of difference in healthcare-seeking behavior was also identified. This may increase the understanding of the inequalities in healthcare-seeking behavior by stakeholders who want to improve women’s health. It could be a good starting point to spread maximum well-being, recovery, and rehabilitation. Among others, one limitation of this review is that we identified and retrieved only open-access papers published in the English language. Hence, the whole body of relevant studies may not have been covered by the present review.

## Conclusions

The review shows that the level of healthcare-seeking behavior is low in low-income countries, and shows wide disparity across settings. In addition, there is shortage ofstudies that assessed healthcare-seeking behavior. We recommend large-scale and more robust studies with sub-group analysis which will help to better understand the healthcare-seeking behavior in general.

## Electronic supplementary material

Below is the link to the electronic supplementary material.

Additional File 1: PRISMA 2020 Checklist

Additional File 2: Critical appraisal of studies that assessed healthcare-seeking behavior of women with pelvic organ prolapse

## Data Availability

All data supporting this information is included in the main document.
