# Supplementary material for: Inequality in healthcare-seeking behavior among women with pelvic organ prolapse: a systematic review and narrative synthesis
Source: BMC Womens Health. 2023 May 3;23:222. doi: 10.1186/s12905-023-02367-3 (PMC10157925; doi:10.1186/s12905-023-02367-3)
Supplement: Supplementary file 2 — Additional File 2: Critical appraisal of studies that assessed healthcare-seeking behavior of women with pelvic organ prolapse [file 12905_2023_2367_MOESM2_ESM.docx]

**Critical appraisal of studies that assessed healthcare-seeking behavior of women with pelvic organ prolapse** (1)

| Author, year | Q1 | | | | Q2 | | | | Q3 | | | | Q4 | | | | Q5 | | | | Q6 | | | | Q7 | | | | Q8 | | | | Q9 | | | | Overall quality result |
| --- | --- | --- | --- | --- | --- | --- | --- | --- | --- | --- | --- | --- | --- | --- | --- | --- | --- | --- | --- | --- | --- | --- | --- | --- | --- | --- | --- | --- | --- | --- | --- | --- | --- | --- | --- | --- | --- |
|  | Y | N | U | NA | Y | N | U | NA | Y | N | U | NA | Y | N | U | NA | Y | N | U | NA | Y | N | U | NA | Y | N | U | NA | Y | N | U | NA | Y | N | U | NA | From 9(100%) |
| [Adhikari and Ranju](#_ENREF_2)., 2018) | √ |  |  |  | √ |  |  |  | √ |  |  |  |  | √ |  |  | √ |  |  |  | √ |  |  |  | √ |  |  |  |  | √ |  |  | √ |  |  |  | 7/9(77.7%) |
| [Dheresa et al., 2020](#_ENREF_5) | √ |  |  |  | √ |  |  |  |  | √ |  |  | √ |  |  |  | √ |  |  |  | √ |  |  |  | √ |  |  |  | √ |  |  |  | √ |  |  |  | 9/9(100%) |
| ([Shrestha et al., 2014](#_ENREF_10)) | √ |  |  |  |  | √ |  |  |  | √ |  |  |  |  | √ |  |  | √ |  |  | √ |  |  |  | √ |  |  |  |  | √ |  |  | √ |  |  |  | 6/9(66.7%) |
| [Hammad et al., 2018](#_ENREF_6) |  | √ |  |  |  | √ |  |  |  | √ |  |  | √ |  |  |  | √ |  |  |  | √ |  |  |  | √ |  |  |  |  | √ |  |  |  | √ |  |  | 7/9(77.7) |
| ([Brazell et al., 2013](#_ENREF_4)) | √ |  |  |  | √ |  |  |  |  | √ |  |  | √ |  |  |  | √ |  |  |  | √ |  |  |  | √ |  |  |  | √ |  |  |  |  | √ |  |  | 9/9(100%) |
| [Morrill et al., 2007](#_ENREF_9)) | √ |  |  |  | √ |  |  |  | √ |  |  |  | √ |  |  |  | √ |  |  |  | √ |  |  |  |  | √ |  |  |  | √ |  |  |  | √ |  |  | 7/9(77.7%) |
| [Jokhio et al., 2020](#_ENREF_7)) | √ |  |  |  | √ |  |  |  | √ |  |  |  | √ |  |  |  | √ |  |  |  | √ |  |  |  | √ |  |  |  |  | √ |  |  | √ |  |  |  | 8/ (88.8%) |
| [Tehrani et al., 2011](#_ENREF_11)) | √ |  |  |  | √ |  |  |  | √ |  |  |  |  | √ |  |  | √ |  |  |  | √ |  |  |  |  | √ |  |  | √ |  |  |  | √ |  |  |  | 8/9 (88.8%) |

**Key: Y=yes, N=no, U=unclear, NA=not applicable, <60%=low, 60-80%=medium, >80%=high quality**

1. Migliavaca CB, Stein C, Colpani V, Munn Z, Falavigna M. Quality assessment of prevalence studies: a systematic review. Journal of Clinical Epidemiology. 2020;127:59-68.
